# The expression of circulating miR-504 in plasma is associated with *EGFR* mutation status in non-small-cell lung carcinoma patients

**DOI:** 10.1007/s00018-019-03089-2

**Published:** 2019-04-05

**Authors:** Adam Szpechcinski, Mateusz Florczuk, Katarzyna Duk, Aneta Zdral, Stefan Rudzinski, Maciej Bryl, Grzegorz Czyzewicz, Piotr Rudzinski, Wlodzimierz Kupis, Emil Wojda, Dorota Giedronowicz, Renata Langfort, Aleksander Barinow-Wojewodzki, Tadeusz Orlowski, Joanna Chorostowska‐Wynimko

**Affiliations:** 1 Department of Genetics and Clinical Immunology, National Research Institute of Tuberculosis and Lung Diseases, 26 Plocka St., 01-138 Warsaw, Poland; 2 Department of Oncology, E.J. Zeyland Wielkopolska Center of Pulmonology and Thoracic Surgery, Poznan, Poland; 3 Department of Oncology, The John Paul II Specialist Hospital, Kraków, Poland; 4 Department of Surgery, National Research Institute of Tuberculosis and Lung Diseases, Warsaw, Poland; 5 II Department of Lung Diseases, National Research Institute of Tuberculosis and Lung Diseases, Warsaw, Poland; 6 Department of Pathomorphology, National Research Institute of Tuberculosis and Lung Diseases, Warsaw, Poland

**Keywords:** Epigenetics, Molecular diagnosis, Liquid biopsy, Predictive biomarker, Targeted therapy

## Abstract

MicroRNAs (miRNAs), key regulators of gene expression at the post-transcriptional level, are grossly misregulated in some human cancers, including non-small-cell lung carcinoma (NSCLC). The aberrant expression of specific miRNAs results in the abnormal regulation of key components of signalling pathways in tumour cells. MiRNA levels and the activity of the gene targets, including oncogenes and tumour suppressors, produce feedback that changes miRNA expression levels and indicates the cell’s genetic activity. In this study, we measured the expression of five circulating miRNAs (miR-195, miR-504, miR-122, miR-10b and miR-21) and evaluated their association with *EPIDERMAL GROWTH FACTOR RECEPTOR* (*EGFR*) mutation status in 66 NSCLC patients. Moreover, we examined the discriminative power of circulating miRNAs for *EGFR* mutant‐positive and -negative NSCLC patients using two different data normalisation approaches. We extracted total RNA from the plasma of 66 non-squamous NSCLC patients (31 of whom had tumours with *EGFR* mutations) and measured circulating miRNA levels using quantitative reverse transcription polymerase chain reaction (RT-qPCR). The miRNA expression levels were normalised using two endogenous controls: miR-191 and miR-16. We found significant associations between the expression of circulating miR-504 and *EGFR*-activating mutations in NSCLC patients regardless of the normalisation approach used (*p* = 0.0072 and 0.0236 for miR-16 and miR-191 normalisation, respectively). The greatest discriminative power of circulating miR-504 was observed in patients with *EGFR* exon 19 deletions versus wild-type *EGFR* normalised to miR-191 (area under the curve (AUC) = 0.81, *p* < 0.0001). Interestingly, circulating miR-504 levels were significantly reduced in the v-Ki-ras2 Kirsten rat sarcoma viral oncogene homolog (*KRAS*)-mutated subgroup compared to *EGFR*-mutated patients (*p* < 0.0030) and those with *EGFR/KRAS* wild-type tumours (*p* < 0.0359). Our study demonstrated the feasibility and potential diagnostic value of plasma miR-504 expression analysis to distinguish between *EGFR*-mutated and wild-type NSCLC patients. However, quality control and normalisation strategies are very important and have a major impact on the outcomes of circulating miRNA analyses.

## Introduction

Targeted therapy with tyrosine kinase inhibitors (TKIs) against the human epidermal growth factor receptor (EGFR) is currently the most common form of personalised treatment for non-small-cell lung carcinoma (NSCLC). Somatic mutations in exons 18–21 of the *EGFR* gene are the most important predictive molecular markers, and their clinical value in diagnosing NSCLC has been confirmed [1]. In-frame deletions in exon 19 account for 44% of EGFR TK-activating mutations: these involve the deletion of amino acid residues leucine-747 to glutamic acid-749. The predominant single point mutation in exon 21, L858R, accounts for 41% of EGFR TK-activating mutations. Changing glycine-719 (G719) to serine, alanine or cysteine accounts for 10%, whereas a duplication and/or insertion in exon 20 accounts for the remaining 5% of EGFR TK-activating mutations [2]. Deletions in exon 19 and the point mutation L858R are termed ‘classical’ activating mutations. These mutations result in increased EGFR kinase activity, leading to enhanced downstream events. Another point mutation in codon 790 [threonine-790 to methionine (T790M)] of exon 20 of the *EGFR* gene was reported in approximately 50–60% of all patients who acquired resistance to EGFR TKI therapy. This mutation is probably acquired during treatment, because it is rarely detected de novo in tumours from untreated patients. *EGFR* mutations occur in approximately 10–20% of Caucasian and 30–50% of Asian NSCLC patients. In contrast to standard chemotherapy, EGFR-TKIs selectively inhibit tumour cell growth by targeting the intracellular EGFR domain. Targeted therapy using TKIs, which bind to EGFR reversibly (e.g., erlotinib, gefitinib) or irreversibly (e.g., afatinib, osimertinib), is the standard personalised treatment for NSCLC in eligible patients [3]. However, most patients develop some resistance to EGFR-TKIs after 9–13 months of treatment.

NSCLC is a challenging diagnostic target due to the substantial diversity of neoplastic clones within tumours, and the difficulties associated with obtaining sufficient quantities of diagnostic material suitable for pathological and molecular evaluation [4]. Moreover, genetic alterations within the tumours change the molecular marker profile during the course of the disease. Currently, most advanced NSCLC patients are diagnosed using biopsies or cytology specimens, due to a lack of availability of resected tumour tissue [5]. However, in many patients, adequate diagnostic sampling is problematic, both de novo and post-EGFR therapy. This is reflected in published data from major clinical trials on EGFR-TKI efficacy, which show that tissue specimens suitable for molecular testing were obtained from fewer than 50% of patients [6–8]. Plasma cell-free DNA (cfDNA) as a liquid biopsy might be a valuable surrogate for detecting tumour-specific alterations in NSCLC patients [9]. The advantages of using cfDNA for tumour mutation detection include: (1) non-invasive specimen collection; (2) availability throughout the course of the disease; (3) real-time detection and monitoring of biomarker status and dynamics; and (4) potentially fewer heterogeneity issues compared to tumour tissue testing [10]. In fact, cfDNA-based assessment of *EGFR*-activating mutation status has proven reliable in identifying NSCLC patients for targeted therapies [11]. However, the rate of *EGFR* mutation detection in cfDNA varies, depending on the method used. A large meta-analysis that included 1591 cases tested using different methods reported a pooled sensitivity rate of 64.5% and results were significantly affected by the assay type (*p* = 0.04) [12].

MicroRNAs (miRNAs) are a class of noncoding, endogenous, single-stranded and relatively small RNA molecules (19–22 nucleotides) that play a key role in the regulation of basic cellular processes including differentiation, proliferation and apoptosis [13]. MiRNA molecules can regulate genes at the post-transcriptional level by specifically recognising messenger RNA (mRNA) molecules, based on the degree of complementarity to the targeted sequence [14]. The aberrant expression of particular miRNAs or miRNA families can result in the abnormal regulation of key signalling pathway components in tumour cells. The oncogenic or suppressor activity of miRNAs in carcinogenesis appears to affect the efficacy of targeted therapies, including EGFR-TKIs in lung cancer [15]. The abnormal regulation of gene expression by miRNAs can trigger alternative signalling pathways or activate downstream signalling mediators, bypassing pathways blocked by EGFR-TKIs. MiRNA expression may change dynamically in response to feedback from miRNA levels and the activity of genes targeted by miRNAs, including oncogenes and tumour suppressors. Furthermore, stable forms of tumour-related miRNAs, called circulating miRNAs, can be detected in the peripheral blood of NSCLC patients. Recently, several circulating miRNA signatures were linked to the mutation status of the *EGFR* gene and/or the response to EGFR-TKIs in NSCLC patients [16–18]. The identification of miRNA profiles linked to altered EGFR signalling and the cellular response to EGFR-TKIs may create new opportunities to develop more effective personalised therapy for patients with NSCLC. These miRNA profiles may also significantly increase the efficacy of molecular diagnostics and targeted treatment monitoring.

However, one major challenge is identifying reliable methods for analysing miRNA expression, particularly that of circulating miRNAs in the blood. MiRNA molecules are small, and those from the same family are highly homologous. They occur at low concentrations in body fluids, and standardised methodologies to detect them are lacking. The various commercial reagent kits and systems available differ in detection method and sensitivity [19]. It has also proved difficult to identify the optimal miRNAs to use as NSCLC biomarkers, and errors have frequently been made during the planning stage of experiments. These errors have included poor patient selection and representation of patient groups, incorrect processing and storage of specimens, and no controls for haemolysis in plasma samples. There is also no consensus on the best method to normalise the resulting data, which is why all steps of an analytical procedure must be validated and standardised before any miRNA-based diagnostic method can be implemented in clinical practice.

In this study, we measured the expression of five circulating miRNAs (miR-195, miR-504, miR-122, miR-10b and miR-21) in plasma taken from NSCLC patients and evaluated their association with *EGFR* mutation status in tumour tissue and cfDNA compared to previously reported results. Moreover, we examined the discriminative power of circulating miRNAs to identify *EGFR* mutant‐positive and -negative NSCLC patients, using two different data normalisation approaches.

## Materials and methods

### Blood collection

Blood was collected from consecutive patients with histologically confirmed primary NSCLC (non-squamous subtype), whose tumour tissue specimens were tested for the *EGFR* mutation. The study was reviewed and approved by the local ethics committee. All patients provided written informed consent.

Peripheral blood was collected from each patient before treatment. Blood was collected in 9-mL tubes containing EDTA as an anticoagulant and processed within 1 h. Plasma was separated from the cellular fraction by centrifugation twice at 1000×*g* for 10 min at 4 °C, and stored at − 80 °C for up to 3 months.

### Initial assessment of haemolysis

The level of haemolysis in the plasma samples was provisionally assessed visually using a haemolysis reference chart [20]. Plasma samples showing moderate or gross haemolysis (i.e., those that were pink to dark red in colour, corresponding to haemoglobin concentrations of approximately > 200 mg/dL) were not used.

### DNA extraction and EGFR/KRAS mutation analysis

Genomic DNA was isolated from deparaffinised 5-µm sections of formalin-fixed, paraffin-embedded (FFPE) tissue specimens using the Cobas DNA Sample Preparation Kit (Roche Diagnostics GmbH, Mannheim, Germany). CfDNA was extracted from 2 mL plasma aliquots using the Cobas cfDNA Sample Preparation Kit (Roche Diagnostics GmbH), in accordance with the manufacturer’s instructions. The mutation status of *EGFR* and v-Ki-ras2 Kirsten rat sarcoma viral oncogene homolog (*KRAS*) in cfDNA was analysed by quantitative polymerase chain reaction (qPCR) using the Cobas EGFR Mutation Test v2 (IVD), the Cobas KRAS Mutation Test v2 (LSR) and the Cobas z480 Instrument (Roche Diagnostics GmbH). The Cobas platform can identify 42 different mutations that may be present in exons 18–21 of the *EGFR* gene and 28 mutations that may be present in exons 2–4 of the *KRAS* gene. The Cobas EGFR test can detect *EGFR* mutations at a mutation level of at least 5% in DNA isolated from FFPE tissue, whereas in plasma, the limit of detection ranges from 10 to 100 copies of mutated cfDNA per millilitre. The Cobas KRAS test can reliably detect *KRAS* variants at ≥ 1% mutation in FFPE and ≤ 75 copies of the mutant allele in a wild-type background of 64,000 copies per millilitre of plasma.

### Immunohistochemical analysis of ALK gene rearrangements

Glass slides with 4-µm FFPE sections were stained immunohistochemically using the D5F3 rabbit monoclonal antibody against human anaplastic lymphoma kinase (ALK) protein, in accordance with the manufacturer’s instructions, and visualised using the OptiView enhanced detection and amplification system and the automated Ventana BenchMark GX Slide Staining System (Roche Diagnostics International Ltd., Basel, Switzerland). Sections were counterstained with haematoxylin and eosin. Human ganglion cells were used as the internal positive control for all specimens. Staining results were evaluated independently by two trained pathologists using the Ventana binary system, which classified strong granular cytoplasmic staining in any tumour cells as a positive result, and the absence of strong granular cytoplasmic staining as a negative result (Ventana Medical Systems, Tucson, AZ, USA).

### MiRNA extraction and spike-in controls

Total circulating RNA was extracted from 100 µL of plasma using QIAzol Lysis Reagent (Qiagen GmbH, Hilden, Germany) and the miRNeasy Mini Kit (Qiagen GmbH), in accordance with the manufacturer’s instructions. A total of 1 µL of synthetic UniSp6 RNA spike-in template, provided with the miRCURY LNA Universal cDNA synthesis kit (Exiqon A/S, Vedbaek, Denmark), was added to each plasma sample treated with QIAzol to monitor the quality of miRNA extraction, reverse transcription and qPCR.

### MiRNA expression measurement using RT-qPCR

The circulating miRNA expression was measured by RT-qPCR. The extracted total circulating RNA was reverse-transcribed into complementary DNA (cDNA) using the miRCURY LNA Universal cDNA Synthesis Kit (Exiqon A/S). Tubes containing 6 µL of RNA template, reaction buffer, primers and reverse transcriptase were incubated for 60 min at 42 °C, followed by 5 min at 95 °C, and cooled to 4 °C in a thermal cycler. The cDNA was diluted 20-fold with water before qPCR amplification.

The qPCR amplification was performed using miRNA sequence-specific miRCURY LNA PCR primers and ExiLENT SYBR Green MasterMix Kit (Exiqon A/S). The qPCR was carried out in 96-microwell plates using the LightCycler 480 II instrument (Roche Diagnostics GmbH). The qPCR cycling conditions were: 95 °C for 10 min, followed by 40 cycles of 95 °C for 10 s and 60 °C for 1 min. The threshold for all samples was set at the same value. Moreover, a melting curve was analysed for each sample to verify the specificity of the amplification products. Each sample was tested in duplicate. In blank samples, the cDNA was replaced by an equivalent volume of water. The targeted miRNA sequences are shown in Table 1.

**Table 1:** Characteristics of the microRNA (miRNA) sequences analysed in this study

Mature miRNA ID	Gene family	Sequence of mature miRNA within the stem loop (5′ → 3′)	miRBase accession number
hsa-miR-195-5p	mir-15	UAGCAGCACAGAAAUAUUGGC	MIMAT0000461
hsa-miR-504-5p	mir-504	AGACCCUGGUCUGCACUCUAUC	MIMAT0002875
hsa-miR-122-5p	mir-122	UGGAGUGUGACAAUGGUGUUUG	MIMAT0000421
hsa-miR-10b-5p	mir-10	UACCCUGUAGAACCGAAUUUGUG	MIMAT0000254
hsa-miR-21-5p	mir-21	UAGCUUAUCAGACUGAUGUUGA	MIMAT0000076
hsa-miR-16-5p	mir-15	UAGCAGCACGUAAAUAUUGGCG	MIMAT0000069
hsa-miR-191-5p	mir-191	CAACGGAAUCCCAAAAGCAGCUG	MIMAT0000440
hsa-miR-451a	mir-451	AAACCGUUACCAUUACUGAGUU	MIMAT0001631
hsa-miR-23a-3p	mir-23	AUCACAUUGCCAGGGAUUUCC	MIMAT0000078

### Final assessment of haemolysis

Each cDNA sample was again tested for haemolysis using a qPCR assay [21]. The level of the erythrocyte-specific miRNA (miR-23a-3p) was related to a miRNA known to be unaffected by haemolysis (miR-451a), allowing outlier samples to be identified and eliminated using the following formulae: delta *C*_t_ (miR-23a–miR-451) > 5, which suggests possible erythrocyte miRNA contamination; and delta *C*_t_ (miR-23a–miR-451) > 7–8, which suggests a high likelihood that haemolysis will affect the data. All samples with delta *C*_t_ (miR-23a–miR-451) > 5 were excluded from the analysis.

### Statistical analyses

The raw data from the RT-qPCR analysis are expressed as mean ± standard deviation (SD) of the qPCR *C*_t_ values for each sample duplicate. The stability of miRNA expression was evaluated using RefFinder, a web-based comprehensive tool integrating the computational programs geNorm [22], NormFinder [23], BestKeeper [24], and the comparative ΔΔ*C*_t_ method [25] to compare and rank the tested genes according to their overall variability among the samples. Based on the rankings from each program, an appropriate weight was assigned to each miRNA and the geometric mean of the weights was calculated to obtain the overall final rankings [26]. The expression levels of the miRNAs tested (miR-122, miR-195, miR-504, miR-10b, and miR-21) were normalised relative to the expression level of each of two endogenous miRNA controls (miR-16 and miR-191). The normalised expression levels of the five miRNAs tested were calculated as Δ*C*_t_s, where ∆*C*_t_ = *C*_t_ (miRNA of interest) − *C*_t_ (endogenous control miRNA), and characterised by their mean ± SD and medians (range), and the coefficient of variance (%CV). The normality of the data was assessed using the Shapiro–Wilk test, and the nonparametric Mann–Whitney *U* test was used to compare the means. Receiver operating characteristic (ROC) curves were constructed to evaluate the diagnostic capacity of circulating miRNAs to predict the *EGFR* mutation status of NSCLC patients [27, 28]. A *p* value < 0.05 was considered statistically significant. All statistical analyses were performed using Statistica (ver. 11; StatSoft, Inc., Tulsa, OK, USA) and MedCalc (Ostend, Belgium) software.

## Results

### Study population

In total, 66 blood samples from NSCLC patients met the quality criteria regarding lack of haemolysis, known *EGFR* mutation in tumour tissue and paired cfDNA. NSCLC histological subtypes were determined according to the World Health Organization’s classification [29]. A total of 56 of the 66 NSCLC patients (85%) had been diagnosed with adenocarcinoma (ADC), 1 patient (2%) had been diagnosed with large cell carcinoma (LCC), 2 patients (3%) with a mixed histology pattern, and 7 patients (10%) with a ‘not otherwise specified’ subtype. NSCLC pathological stages were defined according to the tumour node metastasis (TNM) International Staging System [30]. There were 38 patients (58%) with resectable NSCLC (stages I–IIIA) and 28 patients (42%) with advanced metastatic disease (stages IIIB–IV). No patients had received preoperative chemotherapy or radiotherapy. In total, 31 of the 66 patients (47%) had *EGFR*-activating mutations in their tumour tissue and the remaining 35 patients had *EGFR* wild-type tumours. Among the *EGFR* wild-type group, 12 patients had *KRAS* mutations, 2 had *ALK*-rearrangements and 21 patients had *EGFR*/*KRAS*/*ALK* wild-type tumours. All the patients were Caucasians. The demographic and clinical characteristics of the patients are summarised in Table 2.

**Table 2:** Demographic and clinical characteristics of the study population

Characteristics	*N*		Percent (%)
NSCLC patients	66		100
Median age (years), range		66 (49–88)	
Sex			
Male	37		56
Female	29		44
Histology (WHO)			
ADC	56		85
NOS/other	10		15
Stage (TNM)			
Localized (I–IIIA)	38		58
Metastatic (IIIB–IV)	28		42
EGFR status in tumour			
Mutated	31		47 (100)
Exon 19 deletions	16		(52)
Exon 21 L858R	13		(42)
Other	2		(6)
EGFR wild-type	35		53 (100)
KRAS-mutated	12		(34)
Exon 2 substitutions	10		
Other	2		
KRAS wild-type	23		(66)
ALK-positive (IHC)	2		
EGFR/KRAS/ALK wild-type	21		

### *EGFR* and *KRAS* mutation status of cfDNA

The *EGFR* and *KRAS* mutation status of cfDNA from NSCLC patients was evaluated by qPCR assay using the Cobas platform (Roche Diagnostics GmbH). In total, 14 of the 31 patients (45%) with *EGFR*-activating mutations in their tumour tissue also had mutations in their plasma cfDNA. No *EGFR* mutations were found in plasma cfDNA from patients with localised NSCLC. In the advanced NSCLC subgroup, 14 of the 17 patients (82%) had the same *EGFR*-activating mutation in both tumour tissue and paired plasma. No *EGFR* mutations were detected in cfDNA from plasma taken from the 35 NSCLC patients with *EGFR* wild-type tumours, regardless of the clinical stage of the disease. The 5 out of 12 patients (42%) with *KRAS*-mutated tumours had the same variant in their plasma cfDNA (two patients had localised NSCLC and three patients had advanced disease). No *KRAS* mutations were found in plasma from patients with *KRAS* wild-type tumours.

### Quality control of plasma samples and miRNA expression analysis

**Fig. 1:**
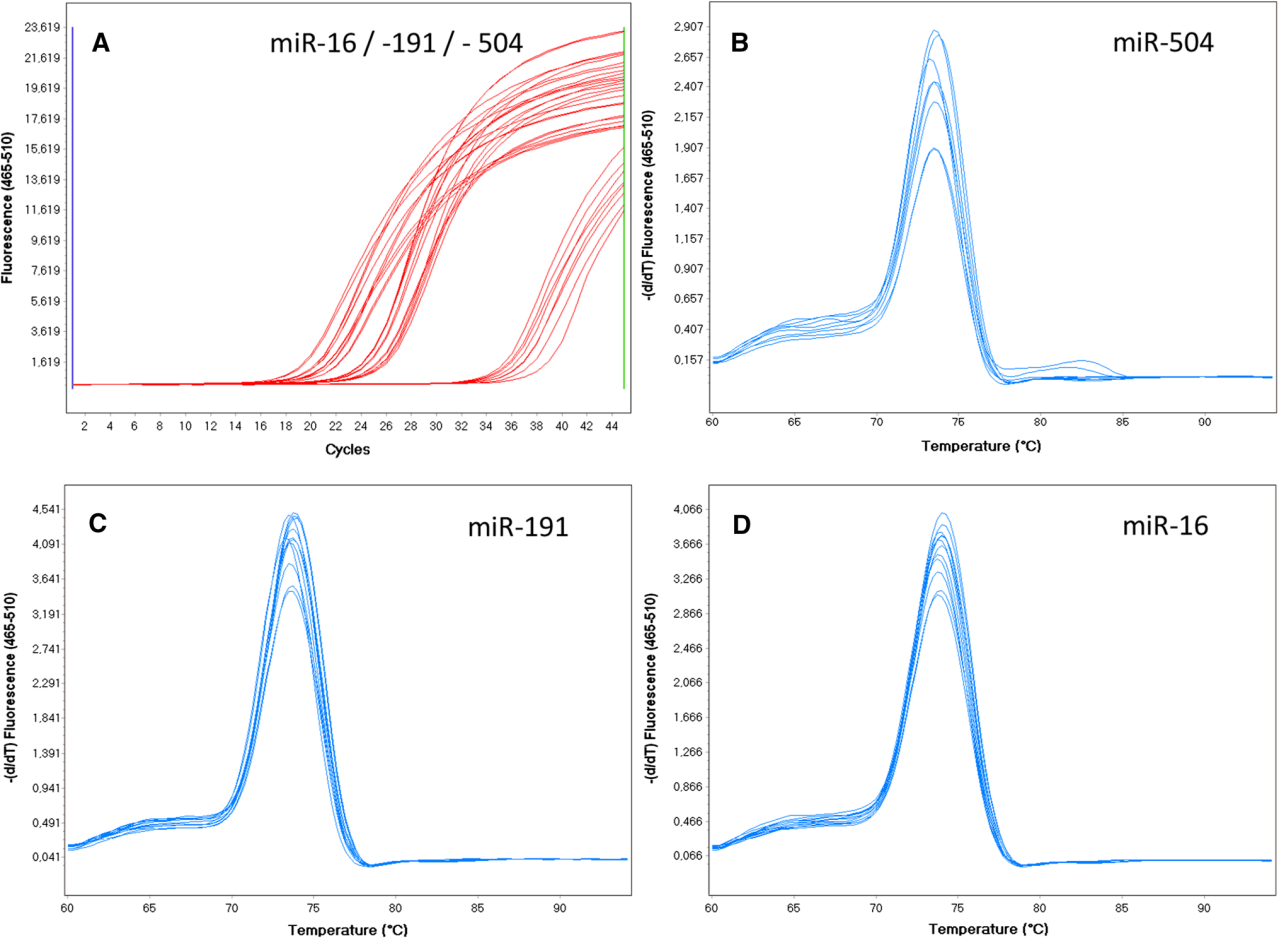
The quantitative polymerase chain reaction (qPCR) amplification curves (**a**) and melting curves (**b**–**d**) for miR-504, miR-16 and miR-191 are shown. The peak at 74 °C in the melting curve corresponds to a particular qPCR amplification product

The level of haemolysis in plasma samples was assessed by RT-qPCR using the delta *C*_t_ (miR-23a–miR-451) formula. The 66 plasma samples included in the study had a mean ± SD delta *C*_t_ (miR-23a–miR-451) of 1.15 ± 1.94 and a median of 1.05 (range − 6.41 to 4.95), which was below the cut-off value of 5.0. The synthetic UniSp6 RNA spike-in template was added to each plasma sample to monitor the quality of miRNA extraction, reverse transcription and qPCR. The samples had highly uniform levels of UniSp6 expression (mean ± SD, *C*_t_ = 19.96 ± 0.87) and relatively low variability among the *C*_t_ values (%CV = 4.36%) indicating good repeatability of sample processing and analysis. Using RT-qPCR with SYBR Green detection to analyse miRNA expression had one major advantage over other techniques: for each sample, amplification product specificity was confirmed by the melting curve analysis. In our study, all the samples had a single melting curve peak at 74 °C, corresponding to a specific qPCR amplification product (Fig. 1). This confirmed that the LNA primers were highly specific and that there were no nonspecific amplification products. The *C*_t_ values of the duplicate samples had very low SDs, ranging from 0.00 to 1.03. None of the blank samples showed any amplification.

### Expression levels of circulating miRNA across all samples tested

**Fig. 2:**
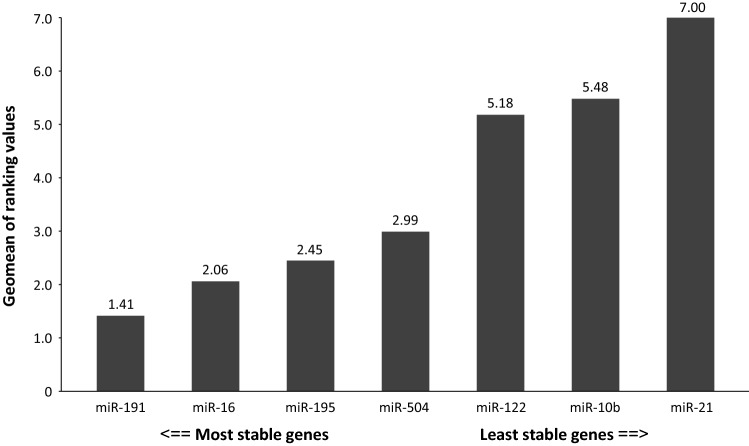
The stability of microRNA (miRNA) expression across the plasma samples was evaluated by RefFinder. The rankings from the major computational programs (geNorm, NormFinder, BestKeeper, and the comparative ΔΔ*C*_t_ method) were used to assign an appropriate weight to each individual miRNA and the geometric means of the weights were calculated to generate overall final rankings. The most stable gene was miR-191

The RT-qPCR results showed that the most abundant miRNA species were miR-16 (mean ± SD *C*_t_ = 18.67 ± 1.47) and miR-21 (20.01 ± 1.61), whereas miR-504 was the least abundant miRNA species (35.02 ± 1.57) in plasma samples. The expression stability of the miRNA species was evaluated using RefFinder. This comprehensive algorithm, described above, selected miR-191 as the most stable gene (comprehensive ranking value of 1.41) and miR-122, miR-10b, and miR-21 as the least stable genes (comprehensive ranking values of 5.18, 5.48 and 7.00, respectively). MiR-16, miR-195 and miR-504 showed moderate variability of expression across the plasma samples (comprehensive ranking values of 2.06, 2.45 and 2.99, respectively; Fig. 2). The expression levels of five tested miRNAs, miR-195, miR-504, miR-122, miR-10b and miR-21, were then normalised using the two endogenous miRNA controls: miR-191 and miR-16. The mean ∆*C*_t_ values for the miRNAs tested are shown in Table 3. A Shapiro–Wilk analysis confirmed that the variables deviated significantly from a normal distribution (*p* < 0.05). Therefore, the miRNA expression results were further compared using nonparametric Mann–Whitney *U* and Kruskal–Wallis tests.

**Table 3:** The normalised expression levels of circulating microRNAs (miRNAs) in plasma relative to the *EPIDERMAL GROWTH FACTOR RECEPTOR* (*EGFR*) and *v*-*Ki*-*ras2 Kirsten rat sarcoma viral oncogene homolog* (*KRAS*) mutation status of non-small-cell lung carcinoma (NSCLC) patients and the endogenous control miRNAs used

Candidate biomarker miRNA	Mutant *EGFR*		Wild-type *EGFR*		Group comparison (*p* values)	
	Normalized expression level (mean ± SD ∆*C*_t_)		Normalized expression level (mean ± SD ∆*C*_t_)			
	miR-191	miR-16	miR-191	miR-16	miR-191	miR-16
miR-195	7.55 ± 0.84	11.36 ± 1.13	7.38 ± 0.87	11.30 ± 1.06	0.1448	0.9386
miR-504	13.24 ± 1.40	17.01 ± 1.54	12.15 ± 1.22	16.10 ± 1.13	0.0072	0.0236
miR-122	3.57 ± 2.48	7.36 ± 2.38	2.96 ± 2.08	6.88 ± 2.40	0.1857	0.3965
miR-10b	7.96 ± 1.62	11.63 ± 1.46	7.29 ± 2.06	11.05 ± 2.05	0.1553	0.3887
miR-21	− 2.19 ± 0.43	1.49 ± 0.84	− 2.33 ± 0.73	1.43 ± 1.03	0.6882	0.7224

Candidate biomarker miRNA	Mutant *KRAS*		Wild-type *EGFR/KRAS*		Group comparison (*p* values)	
	Normalized expression level (mean ± SD ∆*C*_t_)		Normalized expression level (mean ± SD ∆*C*_t_)			
	miR-191	miR-16	miR-191	miR-16	miR-191	miR-16
miR-195	7.68 ± 1.11	11.58 ± 0.76	7.23 ± 0.70	11.15 ± 1.17	0.0854	0.0630
miR-504	11.35 ± 1.18	15.19 ± 1.00	12.43 ± 1.13	16.44 ± 1.00	0.0359	0.0054
miR-122	3.04 ± 0.94	6.94 ± 1.46	2.92 ± 2.50	6.84 ± 2.80	0.4762	0.5901
miR-10b	7.33 ± 1.70	10.95 ± 1.64	7.27 ± 2.26	11.10 ± 2.26	0.8213	0.8757
miR-21	− 2.53 ± 0.97	1.08 ± 1.37	− 2.22 ± 0.57	1.62 ± 0.78	0.6390	0.4141

A nonparametric Mann–Whitney *U* test was used to compare the mean expression levels among groups. A *p* value < 0.05 was considered statistically significant

### Expression levels of circulating miRNA relative to EGFR mutation status

**Fig. 3:**
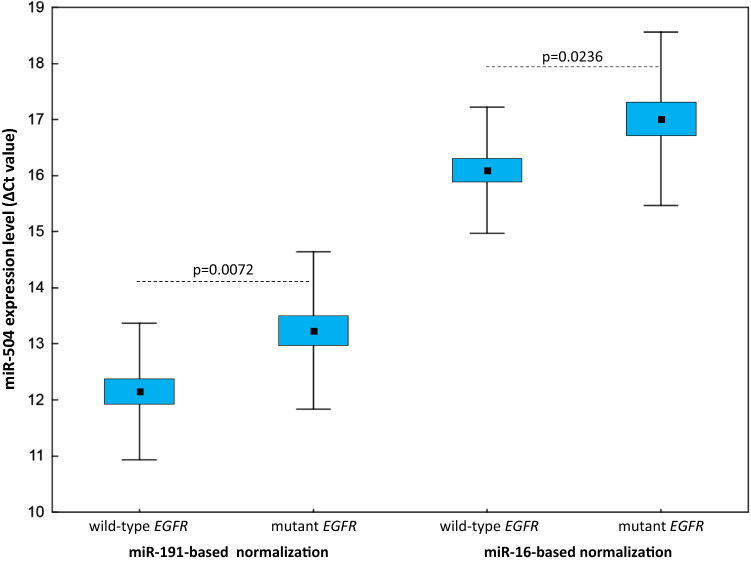
Circulating miR-504 expression levels in epidermal growth factor receptor (EGFR)-mutated and wild-type EGFR patients with miR-191-based and miR-16-based normalisation (Δ*C*_t_ values). The middle mark, box, and whiskers in each plot represent the mean, standard error of the mean, and standard deviation (SD), respectively

NSCLC patients with *EGFR*-activating mutations in their tumours had significantly higher plasma levels of miR-504 compared to those with wild-type *EGFR*, regardless of the normalisation approach used (*p* = 0.0072 for miR-191-normalised data; *p* = 0.0236 for miR-16-normalised data; Fig. 3). When the NSCLC patients were grouped according to the presence of the *EGFR* mutation in cfDNA, only the miR-191-normalised data showed a significant difference (*p* = 0.0122), whereas there was a clear trend approaching significance for the results normalised using miR-16 (*p* = 0.0688). None of the other miRNAs tested showed significant expression level differences when the two NSCLC patient subgroups were compared.

### Expression levels of circulating miRNA relative to KRAS mutation status

**Fig. 4:**
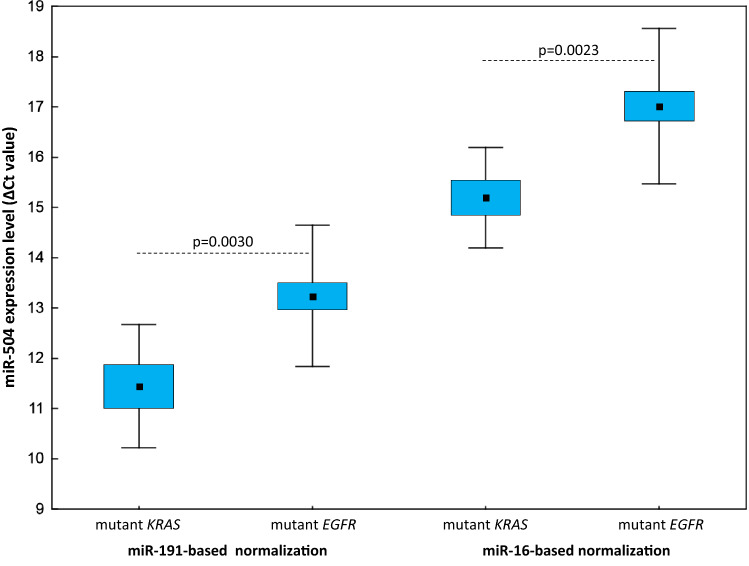
Circulating miR-504 expression levels in *EGFR*-mutated versus *EGFR* wild-type/*v*-*Ki*-*ras2 Kirsten rat sarcoma viral oncogene homolog* (*KRAS*)-mutated patients with miR-191-based and miR-16-based normalisation (Δ*C*_t_ values). The middle mark, box, and whiskers in each plot represent the mean, standard error of the mean, and SD, respectively

Among the *EGFR* wild-type patients, 12 had *KRAS* mutations in their tumours. The same variants were detected in the plasma cfDNA of five of these patients. Plasma miR-504 levels were significantly lower in the *KRAS*-mutated group than in patients with *EGFR* mutations (*p* = 0.0030 for miR-191-normalised data; *p* = 0.0023 for miR-16-normalised data; Fig. 4) and patients with *EGFR/KRAS* wild-type tumours (*p* = 0.0359 for miR-191-normalised data; *p* = 0.0054 for miR-16-normalised data; Table 3), regardless of the normalisation approach used. Significant differences in miR-504 plasma levels were not attributable to the presence of *KRAS*-mutated cfDNA in plasma. None of the other miRNAs tested showed significant expression level differences between NSCLC patient groups separated according to *KRAS* mutation status. Because only two cases with ALK-positive tumours were identified in the *EGFR/KRAS* wild-type group, these data were not statistically analysed.

### Diagnostic power of circulating miR-504 to predict EGFR mutation status

**Fig. 5:**
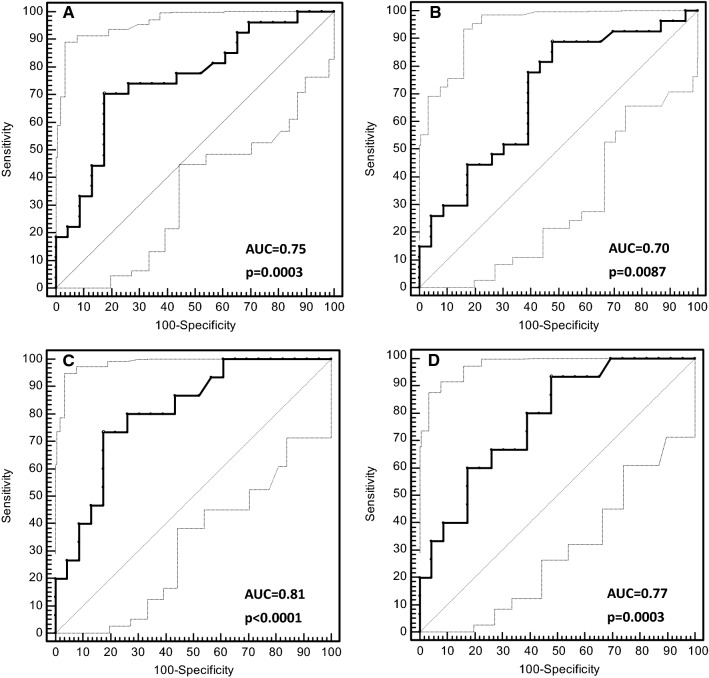
A receiver operating characteristic (ROC) curve analysis was used to evaluate the diagnostic capacity of circulating miR-504 expression to predict *EGFR* mutation status in non-small-cell lung carcinoma (NSCLC) patients. Plots **a** and **b** show how miR-504 can distinguish NSCLC patients with *EGFR* mutations from those with wild-type *EGFR*, using endogenous control miRNA for data normalisation (i.e., miR-191 and miR-16). Plots **c** and **d** show how miR-504 can distinguish NSCLC patients with *EGFR* exon 19 deletions from those with wild-type *EGFR*, using the normalisation approach (i.e., miR-191 and miR-16). The 95% confidence interval for the ROC curve is marked by the dashed grey lines. AUC, area under the curve; P, *p* value

A ROC curve analysis was used to evaluate the diagnostic power of circulating miR-504 levels. This showed that NSCLC patients with *EGFR*-mutated tumours could be distinguished accurately from those with wild-type *EGFR*. The area under the curve (AUC) was 0.75 (95% confidence interval (CI) 0.61–0.87; *p* = 0.0003) for the miR-191 normalised data, and 0.70 (95% CI 0.55–0.82; *p* = 0.0087) for the miR-16 normalised data. For the miR-191-based normalisation approach, the test showed 70.37% sensitivity (95% CI 49.8–86.2) and 82.61% specificity (95% CI 61.2–95.0) at the mean ∆*C*_t_ > 12.74 cut-off point (Figs. 5a, b). When the type of *EGFR* mutation was taken into account, the circulating miR-504 test showed the greatest power to discriminate between the patients with *EGFR* exon 19 deletions and those with wild-type *EGFR* (*p* = 0.0290 for miR-191 normalised data; *p* = 0.0142 for miR-16-normalised data; Figs. 5c, d). At the optimal cut-off point of mean ∆*C*_t_ > 12.74, the test sensitivity was 73.33% (95% CI 44.9–92.2), the specificity was 82.61% (95% CI 61.2–95.0) and the AUC was 0.81 (*p* < 0.0001) for the miR-191-normalised data. No significant relationship was observed for the L858R mutation in exon 21 of the *EGFR* gene.

## Discussion

In this study, we measured the expression levels of five circulating miRNAs (miR-195, miR-504, miR-122, miR-10b and miR-21) in the plasma of NSCLC patients and evaluated their association with *EGFR* mutation status. These miRNAs had been pre-selected by other researchers as candidate biomarkers capable of distinguishing between mutant and wild-type *EGFR* NSCLC patients. To date, these miRNA signatures have not been revalidated in an independent study using a different sample set and a well-established methodology.

To the best of our knowledge, this is the first study to measure the expression of circulating miR-504 in the plasma of NSCLC patients and evaluate its association with *EGFR* mutation status in tumour tissue and cfDNA. Previously, Gasparini et al. used a NanoString nCounter assay to identify miR-1253, miR-504 and miR-26a-5p [31]. These miRNAs were used to distinguish mutation-free (wild-type) NSCLC from translocated *ALK*-, mutant *EGFR*-, or mutant *KRAS*-driven NSCLC, showing an accuracy of 0.79 (95% CI 0.67–0.88) and a multiclass AUC of 0.692 in a set of 67 FFPE tissue samples from NSCLC patients. The *EGFR*-mutated NSCLC patients presented significantly higher (approx. 2.5-fold) normalised expression level of miR-504 (but not miR-1253 and miR-26a; 4.1) than those with wild-type *EGFR* (1.7; *p* < 0.0001). A total of 24 patients had wild-type *EGFR* and 11 had mutated *EGFR*. In total, 10 of the 11 patients with mutated *EGFR* had deletions in exon 19. The association between miR-504 (but not miR-26a) expression and mutant *EGFR* was then confirmed in a validated cohort of 105 lung adenocarcinoma tissue samples from The Cancer Genome Atlas: a high level of miR-504 expression was associated with *EGFR* mutations (odds ratio = 2.86; 95% CI 1.07–7.71; *p* = 0.04). MiR-1253 was not included in the validation set.

In our study, the *EGFR*-mutated NSCLC patients had significantly higher (twofold) circulating miR-504 expression levels than those with wild-type *EGFR*, regardless of the RT-qPCR data normalisation strategy (*p* < 0.05). The discriminative power of circulating miR-504 was particularly high for a subgroup of NSCLC patients with *EGFR* exon 19 deletions in their tumours (AUC = 0.81; *p* < 0.0001). This finding is particularly important because numerous clinical studies have demonstrated that NSCLC patients with exon 19 deletions showed significantly longer median progression-free survival when treated with EGFR-TKIs compared to patients with L858R mutations and uncommon or dual mutations [32–35].

In the ROC analysis, circulating miR-504 expression levels showed more than 70% sensitivity and 83% specificity in distinguishing *EGFR*-mutated NSCLC patients from those with wild-type *EGFR*. Obviously, these values are too low for diagnostic application of surrogate miRNA biomarkers. To date, somatic mutations in exons 18–21 of the *EGFR* gene are the only predictive biomarkers for EGFR-TKI treatment outcomes. In our laboratory, the Cobas EGFR Mutation Test (Roche Diagnostics GmbH) showed more than 83% sensitivity and 100% specificity for detecting *EGFR* mutations in plasma cfDNA from 50 advanced NSCLC patients [36]. In contrast, we found no *EGFR* mutations in plasma cfDNA from 14 NSCLC patients with resectable mutant EGFR-positive tumours, or in 3 of the 17 patients (18%) with metastatic disease whose tumours probably did not shed enough DNA for molecular detection. Nonetheless, five of these patients had miR-504 expression levels that were above the cut-off value of > 12.74, which corresponds to a mutant EGFR-positive result. This clearly demonstrates the diagnostic potential of circulating miRNAs in NSCLC patients to supplement the established algorithm based on the liquid biopsy evaluation, although several specific miRNAs would offer better accuracy than a single biomarker [37, 38]. Blood product fractionation to enrich for tumour-specific miRNAs (i.e., those associated with extracellular vesicles) might further increase the diagnostic power of such biomarkers [39].

Approximately 15–25% of patients with lung adenocarcinoma have tumour-associated *KRAS* mutations [40]. In the majority of cases, these mutations are missense mutations, which involve amino acid substitution at positions 12, 13 or 61, resulting in constitutive activation of KRAS signalling pathways. The RAS proteins [KRAS, v‐Ha‐ras Harvey rat sarcoma viral oncogene homolog (HRAS) and neuroblastoma RAS viral oncogene homolog (NRAS)] are central mediators of growth factor receptor signalling and are, therefore, critical for cell proliferation, survival, and differentiation. RAS can activate several downstream effectors, including the PI3K–AKT–mTOR pathway, which is involved in cell survival, and the RAS-RAF-MEK-ERK pathway, which is involved in cell proliferation [41]. Either *KRAS* mutations are not observed if *EGFR*-activating mutations or *ALK* translocations are present in NSCLC patients [42]. Currently, there are no specific anti-KRAS therapies available. However, *KRAS* mutations are negative predictors of the radiographic response to EGFR TKIs; therefore, their molecular analysis is included in the diagnostic algorithm by some clinical laboratories [43]. The original study by Gasparini et al. showed that the tissue expression level of miR-504 was significantly reduced in *KRAS*-mutated tumours (normalised expression level of 1.6) with respect to *EGFR*-mutated tumours (normalised expression level of 4.1; *p* < 0.0001) [31]. It was also reduced in *EGFR/KRAS* wild-type tumours, although not significantly (normalised expression level of 1.7; *p* > 0.05). In our study, we observed a similar association between the levels of circulating miR-504 and *KRAS* mutation status, again suggesting that circulating miR-504 may be used as a predictive biomarker in NSCLC patients.

Approximately 3–7% of lung tumours, mostly adenocarcinomas, harbour *ALK* fusions [42]. The various N-terminal fusion partners, echinoderm microtubule‐associated protein‐like 4 (*EML4*), Kinesin Family member 5B (*KIF5B*) and TRK-fused gene (*TFG*) genes in particular, promote dimerisation and, therefore, constitutive activity of ALK receptor tyrosine kinase. Signalling downstream of ALK fusions results in activation of cellular pathways involved in cell growth and proliferation [44]. *ALK*-rearrangements do not usually overlap with other oncogenic mutations found in NSCLC patients, e.g., *EGFR* or *KRAS* mutations. Clinically, the presence of *EML4*-*ALK* fusion is associated with resistance to EGFR-TKIs [45]. Gasparini et al. found that miR-504 expression in 17 ALK-positive lung tumours (normalised expression level of 1.8) was significantly lower than in 11 *EGFR*-mutated tumours (normalised expression level of 4.1; *p* < 0.05) [31]. In our study, we identified only two NSCLC patients with ALK-rearrangements among those with *EGFR/KRAS* wild-type tumours, so we could not use these data in statistical analyses. However, the mean expression levels of circulating miR-504 in ALK-positive patients (11.41 for miR-191 normalised data; 15.38 for miR-16 normalised data) were clearly lower than in *EGFR*-mutated subjects (13.24 and 17.01, respectively), which is consistent with the trend observed in tumour tissue.

Because few relevant studies have been published, the biological role of miR-504 in NSCLC remains unclear. A recent report suggested that miR-504 may function as a tumour suppressor in NSCLC [46]. MiR-504 was notably downregulated in NSCLC tissues compared to adjacent normal tissues. Lower miR-504 expression positively correlated with lymph node metastasis and advanced TNM stage in patients. Furthermore, upregulation of miR-504 significantly inhibited cell proliferation, cell invasion and epithelial–mesenchymal transition in NSCLC. RT-qPCR, western blotting analysis and luciferase reporter assays confirmed that miR-504 could bind to lysyl oxidase homolog 2 (*LOXL2*) 3ʹ untranslated regions (UTRs) and regulate its expression. Moreover, miR-504 can function as a negative regulator of human p53 by binding to two sites in the p53 3′ UTR [47]. Overexpression of miR-504 decreased p53 protein levels and activity in lung cancer cells (including p53 transcriptional activity), p53-mediated apoptosis, and cell cycle arrest in response to stress; it also promoted the formation of tumours in vivo. In NSCLC cells, loss of p53 is associated with drug resistance to EGFR inhibitors and radiation [48].

Interestingly, miR-504 occurs within an intron of the fibroblast growth factor 13 (*FGF13*) gene, a member of the FGF homologous factor family [49]. FGF13 and miR-504 probably share a common primary transcript. Expression of the *FGF13* locus, including miR-504, was negatively regulated by p53 in NSCLC. The inhibition of miR-504 expression by p53 may define a p53-regulatory negative feedback loop, possibly boosting p53 protein levels further in response to p53-activation signals.

MiR-504 is misregulated in a variety of cancer types, functioning as an oncogenic miRNA or a tumour-suppressive miRNA. In human glioma, miR-504 suppressed cell proliferation and induced cell apoptosis by targeting Forkhead Box P1 (*FOXP1*) [50]. In osteosarcoma, miR-504 directly targeted tumour protein p53-inducible nuclear protein 1 (*TP53INP1*), which enhanced osteosarcoma growth and promoted distant metastases [51]. In addition, miR-504 expression levels are frequently downregulated in both hepatocellular carcinoma (HCC) tissues and cell lines [52]. MiR-504 also functions as a tumour-suppressive miRNA that inhibits the proliferation and invasion of HCC cells by targeting Frizzled-7 (*FZD7*) and inhibiting Wnt/β-catenin signalling. Bioinformatic analyses and dual-luciferase reporter assays confirmed that miR-504 directly targets the 3ʹ UTR of FZD7 mRNA. Furthermore, increasing evidence suggests that the Wnt/β-catenin pathway is abnormally activated in NSCLC and may be an important mediator of drug resistance to EGFR inhibition. β-catenin levels were increased in NSCLC cells with oncogenic *EGFR* mutations, as well as in gefitinib-resistant cells, and inhibition of the Wnt/β-catenin pathway re-sensitised cells to EGFR inhibitors and increased their efficacy in these tumours [53–55]. Recently, Jiang et al. reported that *EGFR*-activating mutations in NSCLC co-occurred with mutations in genes participating in most key signalling pathways and biological processes, including receptors of different classes, key regulators involved in genome and epigenome stability, the PI3K–AKT–mTOR pathway, and the TP53/apoptosis pathway [56]. In the study by Jiang et al., many of the Wnt/β-catenin pathway-related genes linked to oncogenic EGFRs were mutated, and there was more variation in the Wnt/β-catenin pathway than in the TP53/apoptosis and PI3K–AKT–mTOR pathways. Mutations in *RB1*, *JAK2*, *APC*, *JAK3*, *NF1* and *SMAD4* were predominantly associated with activated mutant EGFRs.

A mature miRNA can guide the miRNA-induced silencing complex (miRISC) to targets using sequence complementarity between the miRNA and sequences in the 3ʹ UTRs of cognate mRNAs. However, experiments have shown that targeting can also occur in the 5ʹ UTRs and open reading frames (ORFs) [57]. Hafner et al. found that among the exonic target regions, 50% of the target sites corresponded to coding sequences (CDSs), whereas only 46% corresponded to 3ʹ UTRs [58]. Chi et al. applied a high-throughput approach to isolate Argonaute-bound target sites, which indicated that target sites in the CDSs were as numerous as those located in the 3ʹ UTRs [59]. Canonical mRNA–miRNA interactions can occur at the 5ʹ region of the miRNA, called the seed (nucleotides 2–7). However, biologically important sites that do not respect canonical seed base pairing have been discovered for a number of targets [60]. Recent publications have highlighted both the abundance and functional importance of non-canonical ‘seedless’ miRNA binding sites in transcriptomes. Because of the degenerate nature of miRNAs outside the seed, the critical determinants of non-canonical sites are probably specific to individual miRNAs/target sites.

Accordingly, we analysed the *EGFR* gene sequence for miR-504 binding sites using the STarMirDB database (http://sfold.wadsworth.org/starmirDB.php), which can identify seeded and seedless sites in the 3ʹ UTRs, CDSs and 5ʹ UTRs of a gene [61]. The software predicted multiple binding sites within the 3ʹ UTR sequence (seven seedless sites), CDS (six seeded sites) and 5ʹ UTR sequence (four seedless sites). Interestingly, many single nucleotide variants were found within the predicted binding sites in *EGFR* sequence using the Ensembl genome database (http://www.ensembl.org), which makes the regulation of *EGFR* by miR-504 even more complicated. No canonical binding site for the miR-504 seed was found within the *EGFR* 3ʹ UTR, and no seedless binding within exons 18–21 of the *EGFR* gene was suggested by the bioinformatic analysis. There is strong experimental evidence that some genes are directly targeted by miR-504, including *TP53*, *TP53INP1*, *MDM2*, *VEGFA*, *BAX* and *CDKN1A*. The implications of this for the EGFR-dependent signalling pathways are well documented (miRTarBase, Release 7.0: 15 September 2017).

Although correlations had been reported by previous investigators, we found no significant relationships between *EGFR* mutation status and the expression levels of the other circulating miRNAs tested: miR-195, miR-122, miR-10b and miR-21. Zhang et al. showed that miR-122 was expressed differently in wild-type (*n* = 48) compared to mutant *EGFR* carriers (*n* = 57) with NSCLC (most cases were at the resectable stage; *p* = 0.018) [16]. Overall survival in patients, especially those with advanced cancer and *EGFR* mutations, was associated with plasma miR-195 levels and miR-122 expression. Zhao et al. showed that upregulation of miR-195, miR-122 and miR-21 was significantly associated with *EGFR* mutations in both tumour tissues and plasma from 149 radically resected NSCLC patients (all *p* = 0.000) [18]. The ROC analysis showed that the AUC values for detecting the *EGFR* mutation were 0.7 (*p* = 0.000; 95% CI 0.618–0.782) for miR-195, 0.733 (*p* = 0.000; 95% CI 0.655–0.812) for miR-122, and 0.8 (*p* = 0.000; 95% CI 0.730–0.870) for miR-21. The AUC of an optimum combination of four plasma miRNAs (miR-195, miR-122, miR-125 and miR-21) was 0.869 (*p* = 0.000; 95% CI 0.808–0.930). Shen et al. showed that the expression levels of circulating miR-21 and miR-10b were significantly higher in radically resected NSCLC patients with EGFR mutations (*n* = 60) than in those without such mutations (*n* = 68; *p* < 0.001) [17].

Currently, there is no standard endogenous control for normalising circulating miRNA levels in the blood. In our study, the expression of circulating miRNAs was normalised to the levels of endogenous control miRNAs, miR-191 and miR-16. Using the comprehensive algorithm RefFinder, described above, we identified miR-191 as the most stable gene (comprehensive stability value = 1.41), followed by miR-16, which showed moderate stability (comprehensive stability value = 2.06) among all the miRNAs tested. Peltier and Latham generated miRNA microarray data from dozens of normal and diseased human tissues and revealed ubiquitous and stably expressed normalisation candidates to evaluate using qRT-PCR [62]. In their study, miR-191 showed the highest consistency in its expression level across 13 normal tissues and five pairs of lung cancer/normal adjacent tissues. This miRNAs was statistically superior to the reference RNAs most commonly used in miRNA qRT-PCR experiments, such as 5S rRNA, U6 snRNA, or total RNA. Hu et al. selected miR-484 and miR-191-5p as the most stably expressed miRNAs and successfully used them as normalizers in serum from breast cancer patients [63].

MiR-16 was also used as an endogenous control to normalise expression data in many previous studies. In our study, the miR-16-normalised and miR-191-normalised expression data were similarly associated with *EGFR* mutation status. McDermott et al. demonstrated that combining miR-16 and miR-425 generated more reliable results than using either of these miRNAs alone, or using RNU6 from blood specimens collected from women with breast cancer and healthy volunteers [64]. Zhang et al. measured the levels of miR‑16 and RNU6B using RT-qPCR in plasma samples from 20 NSCLC patients and 20 healthy controls and observed no significant differences between the two groups (miR-16, *p* = 0.158; RNU6B, *p* = 0.557) [65]. However, when they examined the stability of the two endogenous controls, there were no significant differences in miR‑16 expression among the plasma samples kept at room temperature for 0, 2, 4 and 8 h (*p* > 0.05), whereas the expression levels of RNU6B were significantly different after 4 and 8 h (*p* < 0.05). The results from other studies that have analysed circulating miRNAs in patients with different tumour types also suggest that miR-16 is a good endogenous control [66–68]. However, when selecting a miRNA for normalisation, it is crucial to realise that there can be disease-specific differences in expression patterns because of biological variability among different tumour types [69].

Zhang et al. normalised the expression levels of circulating miR-122 and miR-195 using the exogenous spike-in control cel-miR-39 (synthetic *Caenorhabditis elegans* miRNA) [16]. This spike-in method can eliminate some deviations of the experimental process and make the results more reliable. However, this method does not correct for differences in sampling methods and the quality of samples [70]. A major drawback of using spike-in controls is that although the handling of experiments is taken into account, the quality of tissues, body fluids and extracellular vesicle samples are not. Sample age, body fluid collection procedures, experimental preparation, and storage procedures may produce changes in miRNA levels caused by cell lysis or miRNA degradation.

Zhao et al. and Shen et al. normalised the expression of target miRNAs relative to the expression of small noncoding RNA RNU6 genes, which are commonly used as internal controls for miRNA quantification assays [17, 18]. However, RNU6 is not an miRNA and does not reflect the biochemical character of miRNA molecules in terms of their transcription, processing, or tissue-specific expression patterns [70]. In addition, the efficiency of RNU6 extraction, reverse transcription, and PCR amplification may differ from that of miRNAs. Therefore, it may be best to normalise miRNAs using genes from the same RNA class, i.e., miRNAs [71].

The use of different miRNA normalisation strategies may partially explain discrepancies between our study and the three reports described above, concerning the expression of miR-195, miR-122, miR-10b and miR-21 in the plasma of *EGFR*-mutated versus wild-type NSCLC patients. Another important methodological consideration is haemolysis in the plasma samples. We ensured that none of our plasma samples showed signs of haemolysis using a two-step approach that included visual inspection and measuring the relative expression of the erythrocyte-specific miR-451 and the stable miR-23a.

The effects of haemolysis on the expression of circulating miRNAs have been neglected in many studies. Recent studies have shown that haemolysis during blood collection can have a substantial impact on plasma/serum miRNA content. Kirschner et al. profiled the miRNA content of haemolysed and non-haemolysed plasma and red blood cells to generate a profile of circulatory miRNAs that were affected by haemolysis [72]. They found that miR-16, miR-195, miR-122 and miR-21 were likely to be affected by haemolysis, particularly in plasma from cancer patients. In contrast, miR-191 was unaffected by haemolysis in plasma from healthy individuals and cancer patients. Therefore, rigorous quality control of plasma/serum samples is a critical step before measuring circulating miRNA expression levels. Accordingly, various methods may be used to assess the level of haemolysis in plasma/serum, including simple visual inspection (i.e., identification of a visible pink colour), measuring haemoglobin using a biochemical analyser, measuring haemoglobin by spectrophotometry at 414 nm, and comparing the ratio of red blood cell-enriched miR451a with the stable miR-23a-3p [73]. Measuring the relative expression of miR-451 and miR-23a proved to be the most sensitive method and could detect 0.001% haemolysis in serum.

Extensive variation among pre-analytical conditions and detection methods may account for conflicting results with specific miRNA biomarkers. Haemolysis and data normalisation are only two of the factors affecting the reliability and reproducibility of results pertaining to circulating miRNA expression. Other important factors include blood specimen collection procedures, the effects of fasting and the timing of blood sample collection, blood processing, plasma/serum storage times, miRNA extraction techniques, and miRNA detection methods [74–76].

## Conclusions

MiR-504 expression is misregulated in human cancers, including NSCLC. In this study, for the first time, circulating miR-504 levels in plasma from NSCLC patients were measured and compared to previously published results on differential miR-504 expression in tumour tissue relative to the *EGFR* mutation status of NSCLC patients. Here, we demonstrated the feasibility and potential diagnostic value of circulating miR-504 expression in plasma to discriminate between *EGFR*-mutated and *EGFR* wild-type NSCLC, and also between *EGFR*-mutated and *KRAS*-mutated tumours. Significantly, the greatest discriminative power was achieved for NSCLC patients with *EGFR* exon 19 deletions, which are the strongest predictors of EGFR TKI efficacy. In our analysis, the sample quality control and normalisation strategies for the RT-qPCR data were the key to produce reliable results and evaluating the miRNAs. Consequently, we did not confirm the diagnostic capacity of circulating miR-195, miR-122, miR-10b and miR-21 for discriminating between *EGFR*-mutated and wild-type NSCLC, as reported by other groups.

Further research is needed to confirm the utility of miRNAs, either as predictive biomarkers or therapeutic targets for NSCLC. Currently, there is little or no consensus among published miRNA data due to a lack of standardised protocols for miRNA expression analyses and insufficient attention to pre-analytical variables and quality control. Therefore, we believe it is important to reproduce and revalidate the miRNA expression data published by other groups in independent studies.
